# Carbon Nanotube-Based Intumescent Flame Retardants Achieve High-Efficiency Flame Retardancy and Simultaneously Avoid Mechanical Property Loss

**DOI:** 10.3390/polym15061406

**Published:** 2023-03-11

**Authors:** Qi Qu, Jin Xu, Huanhuan Wang, Yinrui Yu, Qianpeng Dong, Xianhua Zhang, Yan He

**Affiliations:** Shandong Engineering Laboratory for Preparation and Application of High-Performance Carbon Materials, College of Electromechanical Engineering, Qingdao University of Science and Technology, Qingdao 266061, China

**Keywords:** NR, nano flame retardant, IFR structure, CNTs, TA

## Abstract

Intumescent flame retardants (IFR) are an excellent solution to the problem of easy combustion of polymers. Still, the negative effect of the addition of flame retardants is the decline of the mechanical properties of polymers. In this context, carbon nanotubes (CNTs) are modified with tannic acid (TA) and then wrapped on the surface of ammonium polyphosphate (APP) to construct a special intumescent flame retardant structure (CTAPP). The respective advantages of the three components in the structure are explained in detail, especially the role of CNTs with high thermal conductivity in the flame retardant system. Compared with pure natural rubber (NR), the peak heat release rate (PHRR), total heat release (THR), and total smoke production (TSP) of the composites proposed with special structural flame retardants are decreased by 68.4%, 64.3%, and 49.3%, respectively, while the limiting oxygen index (LOI) increased to 28.6%. The TA-modified CNTs wrapped on the surface of APP can effectively reduce the mechanical damage caused by the flame retardant to the polymer. To sum up, the flame retardant structure of TA-modified CNTs wrapped on APP can effectively enhance the flame retardant properties of the NR matrix and reduce the negative impact on mechanics caused by adding APP flame retardant.

## 1. Introduction

Natural rubber (NR) is widely used in various fields of national defense and human livelihood, especially in the fields of transportation [1], shock absorption [2] and sealing [3], due to its excellent physical and chemical properties. There is no synthetic rubber that can completely replace the role of NR in production and life. However, flammability is an inherent disadvantage of most polymers, including NR [4], which undoubtedly increases the risk of their use and limits their wide application in production and life [5]. Therefore, improving the flame retardant properties of NR has extensive and far-reaching practical significance [6]. Compared with the existing flame retardants on the market, such as boron-based flame retardants and metal hydroxide flame retardants, intumescent flame retardants (IFR) have better performance in rubber [7].

Acid, carbon and gas sources are the IFR system’s main components [8]. When the substrate undergoes a combustion reaction, the acid source, carbon source and gas source in the IFR system are, respectively, responsible for promoting the dehydration of the substrate into carbon, replenishing amorphous carbon and generating incombustible gas [9]. The continuous generation of non-combustible gas in the small space causes the space to expand and squeeze the amorphous carbon to form a non-combustible carbonaceous layer [10]. The flame-retardant carbonaceous layer blocks the convection between the external oxygen as a combustion-supporting gas and the non-combustible gas generated by combustion inside the environment [11]. Thus, the purpose of interrupting the combustion reaction of the flame-retardant substrate is achieved by creating an internal environment with a high concentration of non-combustible gas [12]. Unfortunately, the flame-retardant carbonaceous layer formed solely by gas expansion and extrusion is weak and fragile [13]. The broken flame retardant carbonaceous layer cannot play the role of blocking gas convection, which significantly reduces its flame retardant effect [14]. At the same time, the original mechanical properties of the rubber matrix decreased significantly with the addition of a large number of flame retardants [7]. Many methods have been proposed to overcome these shortcomings. Li et al. [15] introduced functionalized graphene into NR to synergize with APP. The results showed that introducing functionalized GE effectively enhanced NR’s flame retardancy, smoke suppression and mechanical properties. However, GE and APP are not in direct contact and are cut off by the matrix when acting, and the synergy efficiency is low. Wang et al. [16] prepared double-shell co-microencapsulated ammonium polyphosphate and mesoporous MCM-41 (M(A&M)) by using melamine-formaldehyde resin and organic silicon in situ polymerization. The results show that introducing both flame retardants can improve NR’s flame retardant and mechanical properties. Although they achieve structural mutual contact, the core–shell presence prevents the flame retardant from reacting rapidly when the substrate burns. The leading solution proposed is the organic combination of intumescent flame retardant and nano-flame retardant.

**Scheme 1:**
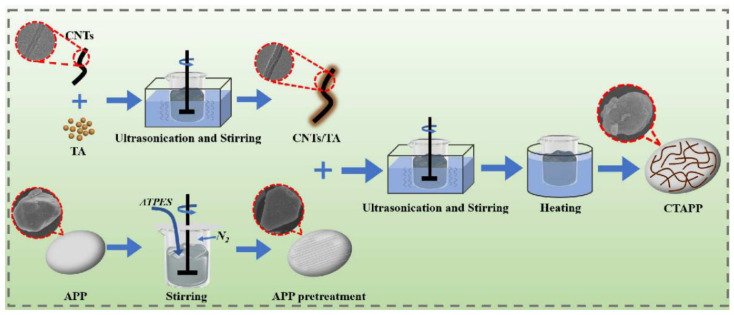
The preparation process for the CTAPP.

Herein, an IFR system, CTAPP, was constructed by wrapping TA-modified CNTs on the surface of APP, as shown in Scheme 1. TA (C_76_H_52_O_46_) has an abundant carbon source and is also an acid source that can produce noncombustible gases during pyrolysis. APP is a traditional acid source that can also cause non-combustible gas. As a medium, CNTs organically combine the carbon source, gas source, and carbon-forming agent into a whole, forming a special IFR structure. This means that the full potential of the flame retardant effect of each component can be reached, the flame retardant performance of the polymer is enhanced, and the negative impact of the flame retardant is reduced. Finally, CTAPP was introduced into the NR latex matrix to explore its fire behavior under high-temperature forced combustion environments, and flammability was investigated with LOI. The gas production process and char residue after combustion were analyzed. The flame retardant mechanism was proposed. The mechanical effects on composites were investigated. 

## 2. Experimental Part

### 2.1. Materials

The carbon nanotube (CNTs, GT-301; diameter: 10–20 nm; length: 5–15 µm; purity > 98%) was generously supplied by Shandong Dazhan Nano Materials Co., Ltd., Shandong, China. Tannic acid (TA), ammonium polyphosphate (APP, polymerization degree ≥ 1000), and (3-Aminopropyl) triethoxysilane (APTES) were purchased from Shanghai Macklin Biochemical Co., Ltd., Shanghai, China. Zinc oxide (ZnO, content at 99.7%), stearic acid (SA, 1801), accelerator M (MBT), and antioxidant (AO, 4020NA) were provided by Shijiazhuang Youlian Chemical Co., Ltd., Hebei, China. The silane coupling agent (Si69) was provided by Dongguan Yuxin Plastic Technology Co., Ltd., Guangzhou, China. The carbon black (CB, N330) was provided by Tianjin Yihuachang New Materials Co., Ltd., Tianjin, China. The sulphur was provided by Jiangsu Huaxing New Materials Technology Co., Ltd., Jinagsu, China. NR latex (solid content: 60 wt%) was provided by Qingdao Double Butterfly Group Co., Ltd., Qingdao, China. All materials are used without any purification.

### 2.2. Preparation of CNTs/TA by TA-Modified CNTs

100 g TA was dissolved into 500 mL deionized (DI) water with magnetic stirring for 30 min. After that, 50 g CNTs was added to the solution, and the mixed solution was ultrasonicated for 30 min. A mass ratio of CNTs:TA of 1:2 is guaranteed. Finally, the CNTs/TA was prepared after magnetic stirring for 6 h. A 10 mL sample was taken, and centrifugation was carried out at a speed of 10,000 r/min using a centrifuge, followed by washing the pellet with deionized water. The centrifugal washing process is repeated three times. Subsequently, the pellet was dried at 60 °C for 3 h to remove excess water and obtain the CNTs/TA powder. The quality ratio of CNTs and TA in the powder was determined using a thermogravimetric analyzer (TGA), which facilitates subsequent analysis.

### 2.3. Preparation of CTAPP by CNTs/TA Wrapped APP

Firstly, 50 phr APP was dissolved into 100 mL absolute ethanol and before magnetic stirring 30 min under N_2_ atmosphere. Additionally, 5 phr ATPES solution dropwise was added in the process of magnetic stirring. After that, the APP pretreatment solution was obtained. Secondly, 12.25 phr CNTs/TA was dispersed into a beaker containing 100 mL ethanol and 100 mL DI water. A mass ratio of APP:CNTs/TA of 4:1 is guaranteed. Additionally, the mixed solution was ultrasonicated for 30 min at room temperature. The pretreatment solution of APP was poured into CNTs/TA and sonicated at 60 °C while being mechanically stirred for 30 min; it was then =transferred to an oil bath for 6 h at the same temperature. Finally, the CTAPP was obtained by centrifugation, rinsing and drying.

### 2.4. Preparation of NR/CTAPP by Adding CTAPP

According to the s formulations of composite in Table 1, the NR/CTAPP were made by adding CB (30 phr) and CTAPP (variable) in turn while performing high-speed mechanical stirring on the NR latex. Glacial acetic acid was slowly added dropwise during low-speed mechanical stirring for flocculation. The NR/CTAPP was cut into small pieces and washed repeatedly with DI water to thoroughly wash off excess glacial acetic acid. Then, the NR/CTAPP was dried at 80 °C for 12 h. The dried NR/CTAPP was added to an internal mixer (Haake, 200 cc) and mixed for 8 min. Antioxidant (1 phr), zinc oxide (5 phr), stearic acid (2 phr), and silane coupling joint agent (3.5 mL) were added to the internal mixer during the mixing process. NR/CTAPP was added to a two-roll mill at 25 °C with the addition of sulfur (1.4 phr) and accelerator (1.2 phr) after standing for 3 h. The mixture was cured in a flat vulcanizer after standing for 12 h.

### 2.5. Characterization

The Fourier transformed infrared (FTIR) spectroscopy was obtained with TA-modified CNTs acquired on a Nicolet iS50 FT-IR infrared spectrometer from 4000 to 400 cm^−1^.

The thermal stability of the CNTs/TA was measured using thermal gravimetric analyzer (TG 209 F3 Tarsus^®^, NETZSCH, Germany) under an oxygen atmosphere (20 mL/min).

The surface morphologies of the APP, CTAPP, and the char residue were observed using scanning electron microscopy (SU8010, Hitachi, Japan) at an acceleration voltage of 10 kV under a low vacuum.

The composition of CTAPP was recorded by X-ray diffraction (XRD) analysis (Rigaku MiniFlex600, Japan) in the range of 5–90°.

The limiting oxygen index (LOI) value was tested on an HC-2C oxygen index apparatus (Nanjing Jiangning Analytical Instrument Co., Ltd., Jiangsu, China.) using ASTM D2863 with a sample dimension of 100 × 5 × 3 mm^3^. Five samples were tested to obtain an average value.

The UL-94 vertical burning rating was obtained using CZF-4 vertical testing apparatus (Nanjing Jiangning Analytical Instrument Co., Ltd., Jiangsu, China.) according to ASTM D3801. The polymeric composites were prepared with the dimension of 130 mm × 13 mm × 3 mm. The test was repeated three times.

Heat and smoke releases of composites during combustion were recorded on a cone calorimeter (Fire Testing Technology, United Kingdom) according to ASTM E1354/ISO 5660. The sample with the dimension of 100 mm × 100 mm × 3 mm was ignited and combusted under 35 kW/m^2^ irradiation.

Mechanical properties were carried out with a Gotech-AI-7000-MGD Testing Machine (Taiwan, China), as per ISO 37-2005 standard.

## 3. Results and Discussion

### 3.1. Characterization of Modified Flame Retardant CTAPP

**Figure 1:**
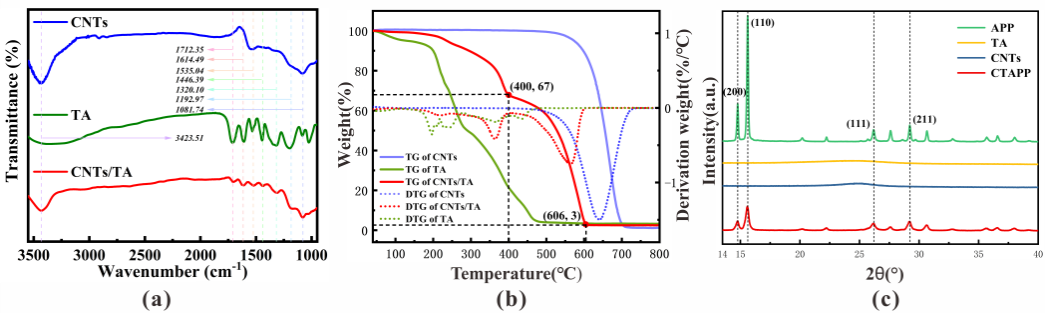
(**a**) The FT-IR patterns of CNTs, TA and CNTs/TA. (**b**) TG and DTG thermograms of APP and CTAPP in O^2^ atmosphere (*V*_*O*_2__:*V*_*N*_2__ = 1:4) at the heating rate of 5 °C/min. (**c**) XRD patterns of APP, TA, CNTs and CTAPP.

FT-IR was used to characterize the surface functional groups of TA-modified CNTs (CNTs/TA) as shown in Figure 1a. It shows several specific adsorption peaks between 1080 cm^−1^ and 1750 cm^−1^ compared with the pristine CNTs. It can be clearly seen that the appearance of the adsorption band at 1750–1080 cm^−1^ is the result of CNTs modified by TA. Additionally, the existence of a characteristic peak at 3423.51 cm^−1^ represents the stretching vibration of the hydroxyl group (-OH) [17]. In the spectrum of TA, the characteristic peaks appear at 1712.35, 1614.49, 1535.04, 1446.39, 1320.10, 1192.97 and 1081.74 cm^−1^ and correspond to the stretching vibration of C=O, C-O and C=C, respectively [18,19]. The differential characteristics of CNTs and CNTs/TA further demonstrate the successful preparation of CNTs/TA. Meanwhile, through comparison, it is found that the FTIR spectra of CNTs/TA are just simple physical superpositions of the FTIR spectra of CNTs and TA, and no new characteristic peak or significant peak shift is observed. This indicates no chemical interaction, but only physical interaction between CNTs and TA during the modification process.

The TGA and DTG curves of CNTs, TA, and CNTs/TA under the O_2_ atmosphere (*V*_*O*_2__:*V*_*N*_2__ = 1:4) are shown in Figure 1b [20,21]. It can be found that the weightlessness process of TA starts at about 50 °C and completes the pyrolysis process at 480 °C. The degradation of CNTs starts at 500 °C and ends at 700 °C. The pyrolysis temperature of TA and CNTs is quite different. The thermal pyrolysis process of CNTs/TA presents an obvious second-order step [22]. CNTs/TA starts weightlessness at 50 °C and is slowly pyrolyzed at 50–400 °C. The pyrolysis process conformed to the 50–480 °C section of the pyrolysis curve of TA. At 400–606 °C, CNTs/TA also undergoes a slow initial and final fast pyrolysis process, which is consistent with the pyrolysis curve characteristics of CNTs at 500–700 °C. Therefore, it can be considered that the pyrolysis temperature range of TA in CNTs/TA is 100–400 °C, the quality ratio is 33%, while the pyrolysis temperature range of CNTs is 400–606 °C, the quality ratio is 64%. It can be determined that the mass ratio of CNTs and TA is 2:1 [23]. Thus, APP:CNTs:TA = 12:2:1 in CTAPP. In addition, it is not difficult to find that the initial pyrolysis temperature and termination temperature of CNTs after modifying TA are significantly reduced. The doping of TA with low pyrolysis temperature in CNTs with high pyrolysis temperature reduces the overall pyrolysis temperature [24]. The analogous combustion reaction can explain this phenomenon, and the low-ignition-point TA, modified onto the high-ignition-point CNTs, plays a particular role in igniting the CNTs/TA [25]. 

**Figure 2:**
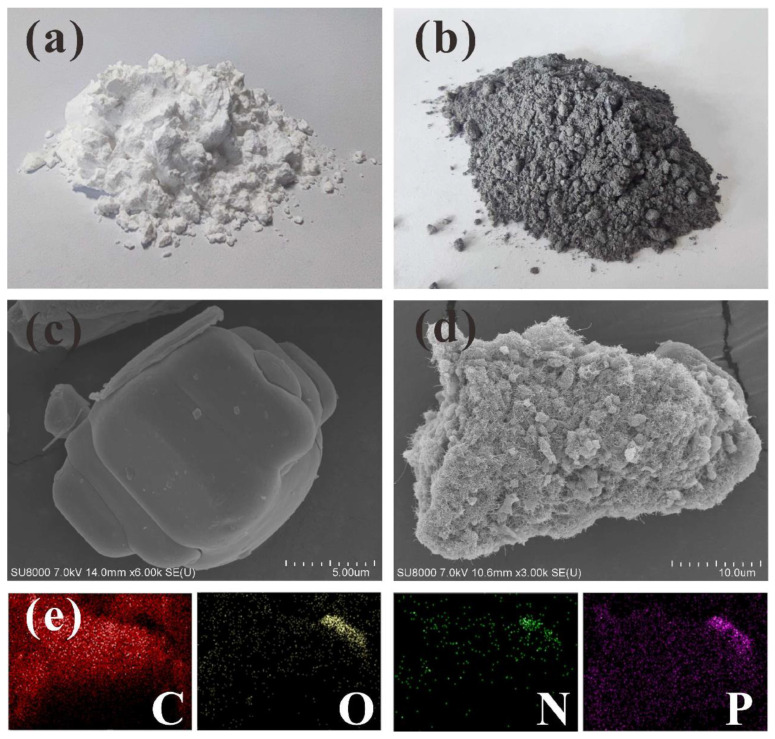
The digital and SEM images of APP and CTAPP: (**a**) macro view digital image of APP; (**b**) macro view digital image of CTAPP; (**c**) microscopic SEM image of APP; (**d**) microscopic SEM image of CTAPP; (**e**) EDS-mapping spectrum of the CTAPP.

The digital and SEM images are shown in Figure 2. The digital and SEM images of APP before and after the modification are shown in Figure 2. In Figure 2a,c are the digital and SEM images of pure APP, respectively, and it can be found that APP is a pure white powder under macroscopy and a block with smooth surface under microscopy. In contrast to the former, Figure 2b,d point out that the CTAPP formed after wrapping with CNTs/TA appears grayish black macroscopically and microscopically as a block with a surface covered by tubular material. In order to confirm that the substance in Figure 2d is indeed the APP wrapped by CNTs, rather than the bulk substance formed by the self-aggregation of pure CNTs, the EDS-mapping spectrum is carried out as shown in Figure 2e. The sample in the field of view in Figure 2d is a more representative sample that is selected, with more CNTs wrapped on its surface and a smooth upper-right part with no CNT wrapping. In Figure 2e, carbon occupies the entire visual space, and the red dots of carbon in the CNTs wrapped on the material’s surface are much denser than those mapped by the surrounding conductive tape. The N, O, and P element patterns show evident regular distribution. On the one hand, the outline of the elemental mapping points is utterly consistent with the SEM image. On the other hand, the upper right corner of the block that is not covered by the modified CNTs can be seen in Figure 2d as the dense and highlighted mapping points of the N, O, and P elements in Figure 2e. The elemental mapping is entirely consistent with the SEM observation image, and it is comprehensively confirmed that the modified CNTs can be successfully wrapped on the APP, and the modified CNTs microencapsulated APP can be realized. Thus, the construction of a synergistic flame retardant structure using CNTs as a mediator was completed. The carbon source TA and the acid source APP are directly contacted to participate in the carbon-forming reaction. The CNTs and the flame-retardant carbonaceous layer are directly contacted to participate in the supporting carbonaceous layer.

The XRD patterns of APP and CTAPP are shown in Figure 1c. It can be found that the diffraction peaks of APP at 14.7°, 15.5°, 26.1°, and 29.1° ] correspond to (200), (110), (111), (211) planes, respectively [26]. CNTs and TA are amorphous materials with no diffraction peaks [27,28]. CTAPP is the product of a synergistic synthesis of three materials. Due to the doping of two amorphous materials, no additional diffraction peaks are added, but the intensity of each diffraction peak is weakened. This phenomenon proves that the modification process does not change the regular periodic arrangement of material particles and does not generate any new substances [29].

### 3.2. Analysis of NR/CTAPP Flame Retardant Properties

#### 3.2.1. Flammability Analysis of NR

The macroscopic phenomena and flame retardant data of the NR/IFR combustion were analyzed, and preliminary exploration of its flame retardant performance was conducted. The LOI value and UL-94 value data of NR/IFR are shown in Table 2. The NR0 is pure NR without any flame retardant. When ignited, it burns rapidly until the combustion is complete, accompanied by the phenomenon of molten droplets, and can ignite the cotton props used for testing, so there are no vertical data for UL-94 [30]. Meanwhile, the LOI value is only 19.6% after testing, thus determining it to be a flammable material. On the basis of NR0, composites (NR1–NR6) are made by adding different kinds and different contents of flame retardants.

**Figure 3:**
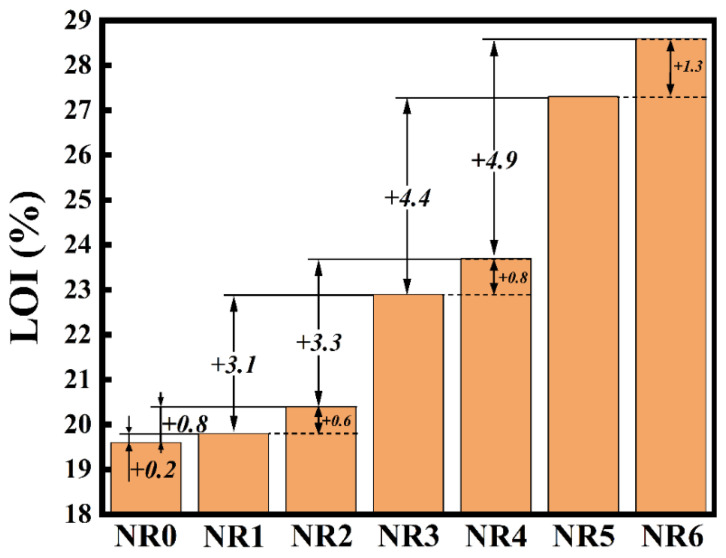
LOI Trend analysis results.

In longitudinal grouping analysis composites NR0, NR1, NR3, NR5, and NR0, NR2, NR4, NR6, it can be concluded that the limiting oxygen index increases with the increase in the addition amount of the two flame retardants. NR6 added with 50 phr CTAPP flame retardant achieves a maximum LOI of 28.6%, and also achieves a V-0 rating in the UL-94 test (when the composite NR6 is ignited twice for 10 s, NR6 can achieve self-extinguishing within 30 s, and no combustion material falls). Meanwhile, by calculating the increase in the limiting oxygen index of the two groups of samples, as shown in Figure 3, we can observe the following. With the increase in the addition amount of flame retardant added, the flame retardant performance provided by each 20 phr flame retardant is the flame retardant efficiency. It is an upward trend, and has not yet reached its peak. In lateral grouping compares composites NR1–2, NR3–4, and NR5–6, it can be found that the flame retardant properties of the composites with the same proportion of flame retardants are significantly different. Comparing NR1 and NR2, the LOI value of NR2 is 0.6% higher than that of NR1, and the latter two groups are 0.8% and 1.3%. For every 20 phr increase in flame retardant, the increase of LOI value, NR/CTAPP, is higher than NR/APP. As expected, both LOI and UL-94 data of the composites have shown that the improvement of the flame retardancy of NR when using TA-modified CNTs wrapped with APP instead of partial APP is quite obvious. Multiple components with special structures in CTAPP have a synergistic flame retardant effect.

#### 3.2.2. Cone Calorimeter Test

**Figure 4:**
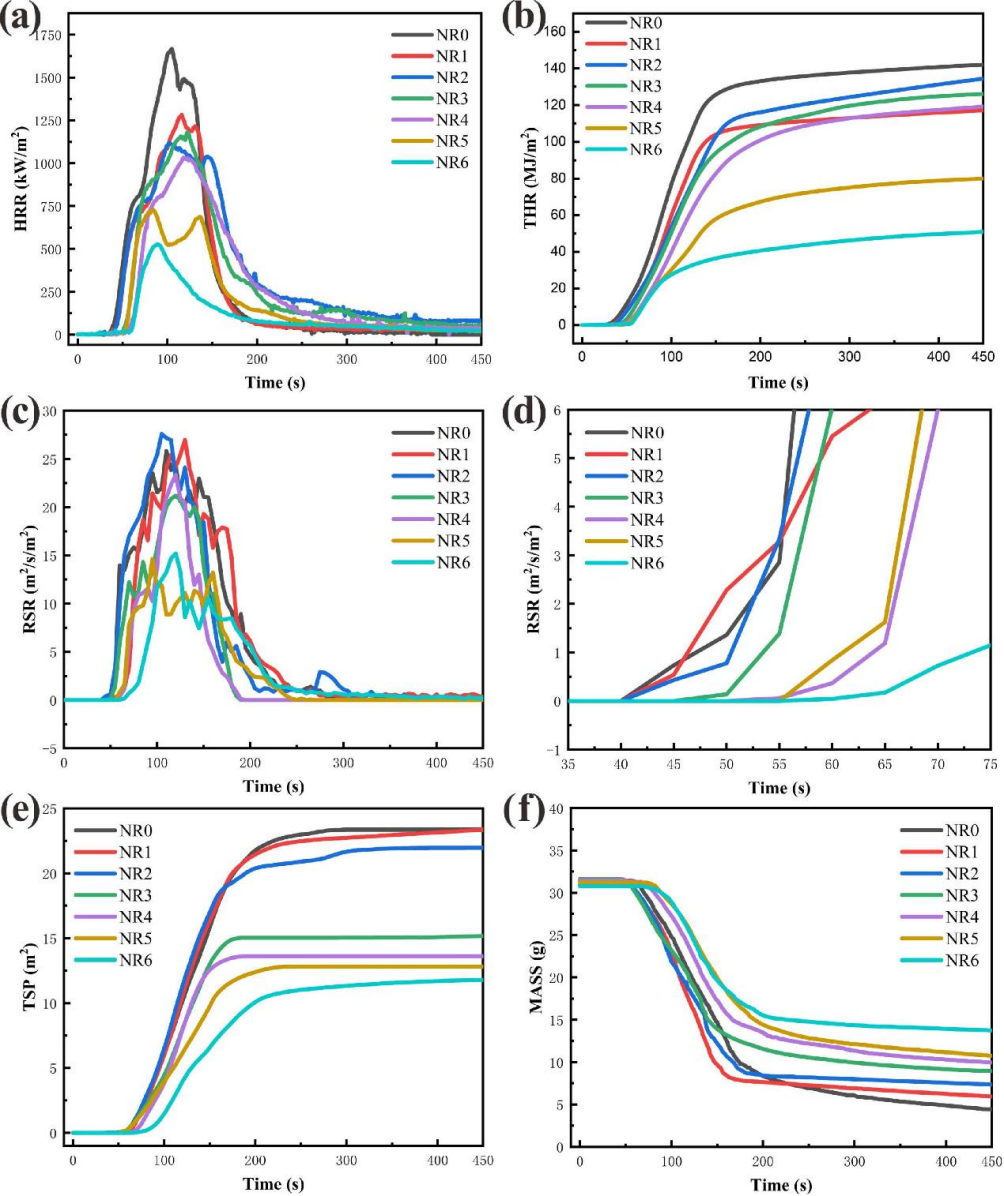
Cone calorimeter test process curve: (**a**) HRR; (**b**) THR; (**c**) RSR; (**d**) enlarged view of RSR; (**e**) TSP; (**f**) specimen mass.

In order to comprehensively characterize the burning behavior of the composites, the cone calorimeter test (CCT) was used to explore burning behavior under the radiation of an external heat source of 35 KW/m^2^ [31]. Experimental results include time to ignition (TTI), the peak heat release rate (PHRR), time to peak heat release rate (TPHRR), total heat release (THR), total smoke production (TSP), and mass. The residue after combustion (MASS) is presented in Table 3. The experimental process is shown in Figure 4.

Heat release rate (HRR) is an essential factor to characterize combustion [12]. In Figure 4a,b, NR0 has a fast burning rate and prominent THR characteristics. The PHRR of NR0–NR6 is gradually decreasing, and the peak value of NR6 is reduced to 527.20 kW/m^2^ at NR6. The law of THR and HRR is similar, and also reaches a minimum value of 50.85 MJ/m^2^ at NR6. It must be pointed out that a small amount of flame retardant was added toNR1–NR4. Although the HRR is effectively reduced, the THR amount is not significantly reduced. However, this still proved that it reduced the burning intensity of the composites, and the effect of NR/CTAPP is better than that of NR/CTAPP with the same amount of flame retardant added. Meanwhile, the fire performance index (FPI) and the fire growth index (FGI) are introduced to characterize the safety hazards of composites, as shown in Table 3. FPI is defined as the quotient of TTI and PHRR [32], and FGI is defined as the quotient of PHRR and TPHRR [33]. Larger FPI and smaller FGI are judged to be more minor security risks., i.e., the larger the TTI, the smaller the PHRR, and the larger the TPHRR. The FPI law of NR0–NR6 in Table 3 is entirely similar to the above HRR law, and reaches a maximum value of 11 10^−2^ m^2^s/kW at NR6. The FGI of NR/CTAPP is smaller than that of NR/APP when the same amount of flame retardant is added. The combined FPI data have shown that CTAPP flame retardants can significantly reduce the fire safety hazards of NR. The flame retardancy index (FRI) is defined and used to measure the effectiveness of flame retardants. It is the ratio of THR × PHRR/TTI between the corresponding thermoplastic composites consisting of pure polymers, and those containing only one type of flame retardant additive. Additionally, flame retardant performance can be characterized using FRI, where FRI < 1 indicates poor performance, 1 < FRI < 10 indicates good performance, and FRI > 10 indicates excellent performance [34]. After the proposal of the FRI, it has gained recognition from a large number of scholars in the field of flame retardancy, and has been used in their articles as a means of characterizing flame retardant performance [35,36,37,38]. The FRI values of NR1-NR5 are all greater than 1 and less than 10, indicating good flame retardancy performance. In addition, the FRI value of NR6 is 15.07, which indicates excellent flame retardancy performance. This largely demonstrates their good synergistic flame retardancy effect.

The toxic gas released by burning polymers is the main factor leading to increased fire damage [39]. In Figure 4c,e, CTAPP flame retardant can effectively reduce the RSR and TSP. The smoke suppression effect of CTAPP flame retardant is better than that of a single flame retardant. As shown in Figure 4d, the growth rate of the smoke production rate of CTAPP flame retardant composites is less than that of APP flame retardant composites within 40–70 s in the initial stage of combustion. This is because the presence of CNTs in CTAPP increases the thermal conductivity of the composite, making the flame retardant participate in the combustion reaction faster. In addition, the low reaction temperature of TA makes CTAPP more sensitive to combustion. The non-combustible gas generated by the thermal decomposition of CTAPP extrudes the amorphous carbon to form a flame-retardant carbonaceous layer. The CNTs support the flame-retardant carbonaceous layer to make it more robust and to trap flame-retardant gases inside the composite. On the one hand, the leakage of various gases generated by thermal decomposition inside NR/IFR is reduced. On the other hand, the flame retardant performance of NR/IFR is effectively improved.

#### 3.2.3. Analysis of Char Residue

**Figure 5:**
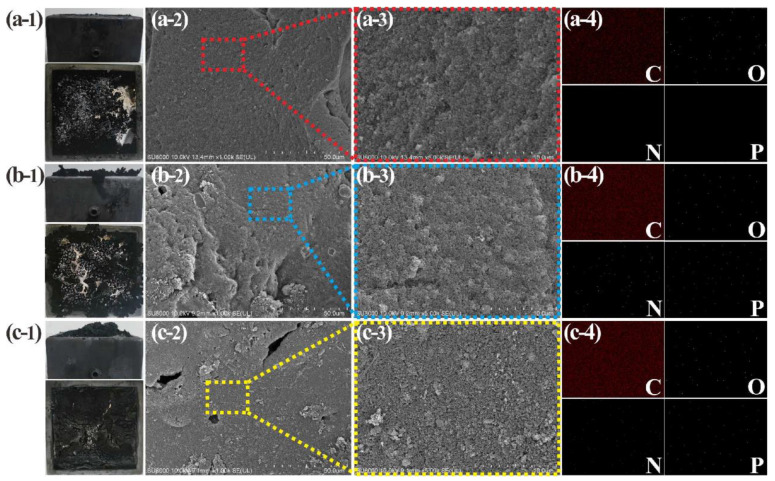
Char residue analysis of NR0–NR2 composites after CCT: digital photos (**a-1**,**b-1**,**c-1**); SEM images (**a-2**,**b-2**,**c-2**,**a-3**,**b-3**,**c-3**); EDS-mapping spectrum after 15 min of scanning (**a-4**,**b-4**,**c-4**).

**Figure 6:**
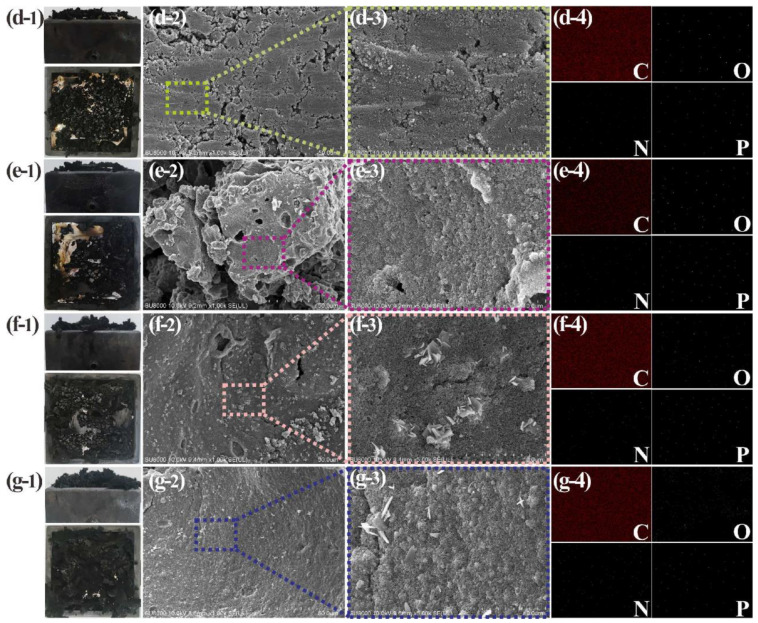
Char residue analysis of NR3–NR6 composites after CCT: digital photos (**d-1**,**e-1**,**f-1**,**g-1**); SEM images (**d-2**,**e-2**,**f-2**,**g-2**,**d-3**,**e-3**,**f-3**,**g-3**); EDS-mapping spectrum after 15 min of scanning (**d-4**,**e-4**,**f-4**,**g-4**).

In order to further analyze the flame retardant behavior of CTAPP flame retardant, the macroscopic and microscopic morphologies of the char residues after the CCT were specially selected for comparative analysis [40]. Figure 5 and Figure 6 contain digital photographs, SEM images, and mapping energy spectra of NR0–NR6. 

In Figure 5a-1, the physical image of the char residue after the combustion of pure NR is shown. The char residue surface is fine and smooth. The char residues of NR1–NR6 composites all have different degrees of expansion. This is caused by the NH_3_-extruded carbonaceous layer formed by thermal decomposition of APP, and the expansion degree is positively correlated with the addition amount of APP. The mass of char residue produced by the combustion of the NR/CTAPP is larger than NR/APP, and it is easier to ensure the original shape of the NR [41]. This feature can be confirmed by comparing three groups of composites with the same proportion of different types of flame retardants. It is most notable for NR1 and NR2 (Figure 5b-1,c-1). The reason that the char residue is sturdy is that the CNTs act as the support of the skeleton. The topography of the CNTs-supported char residue is shown in Figure 5 [42].

The char residue of pure NR is consistent, without impurities of other shapes. The char residues of NR1–NR6 composites have various morphologies. The main feature is the presence of numerous voids or cracks. The reason is that the excessive NH_3_ pressure generated by the pyrolysis of APP leads to the breaking of the carbonaceous layer [43]. Besides, it can be clearly observed that the porosity of NR/CTAPP char residue is less than that of NR/APP char residue. This is because the presence of CNTs reinforces the flame-retardant carbonaceous layer, making it more difficult for the generated gases to break through this barrier. Thereby, the external oxygen environment and the internal environment where various flame retardant gases exist are effectively isolated, thus serving the purpose of improving flame retardant efficiency [44]. It should be pointed out in Figure 6f-2 that there is a large amount of APP remaining; this plays a leading role in flame retardancy. From this, it can be concluded that a small amount of APP can achieve the purpose of flame retardant. In Figure 5, the problem of APP waste is greatly improved, the unutilized APP is reduced, and the flame retardant performance of the composite is enhanced.

In the elemental mapping of pure NR, only C and O fluorescent spots are evenly distributed on it. The elemental mappings of NR1–NR6 are all covered by the fluorescent spots of C, O, N, and P elements. APP supplies the N and P elements. Therefore, compared with the composites with the same amount of flame retardant, the N and O element residues of the composites with only APP are higher than those with CTAPP. It is speculated that the high thermal conductivity of CNTs wrapped on APP’s surface lowers APP’s decomposition temperature and prolongs the decomposition time to make it function more efficiently.

#### 3.2.4. Flame-Retardant Mechanism

**Figure 7:**
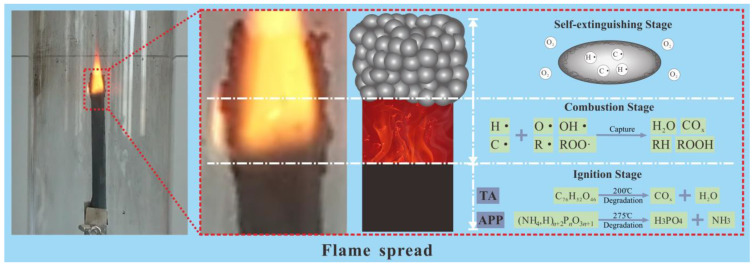
Flame divided into three stages: ignition, combustion, and self-extinguishing.

CNTs are used to organically combine the carbon source TA and the acid source APP to achieve higher flame retardant efficiency. The flame retardant mechanism of the TA-modified CNTs encapsulated APP flame retardant is described in three stages from ignition to self-extinguishment according to flame spread, as shown in Figure 7.

**Figure 8:**
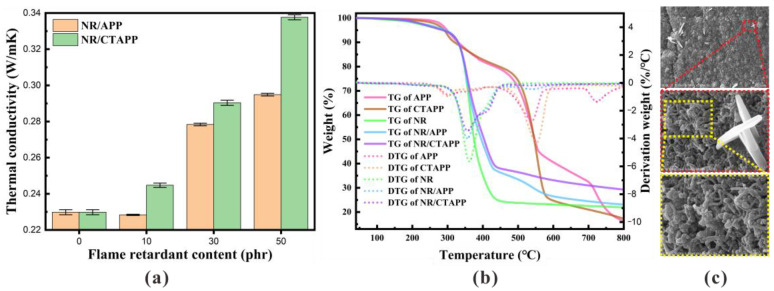
(**a**) Thermal conductivity of composites. (**b**) TGA analysis curves of APP, CTAPP, NR, NR/APP, and NR/CTAPP under nitrogen atmosphere. (**c**) CNTs in residual char residues.

First, the process of the NR/CTAPP just being ignited is defined as the ignition stage. At this stage, the temperature is low, and the CNTs with high thermal conductivity rapidly conduct the heat from the rubber into the CTAPP. TA is slowly pyrolyzed by heating and initially carbonized to prepare for the intumescent flame retardant. The thermal conductivity of the composites is shown in Figure 8a. The thermal conductivity of the composites after the addition of APP has slightly improved. The thermal conductivity of the composites after partial replacement of APP with CNTs/TA is significantly improved. As can be seen in Figure 8b, the rapid thermal decomposition of TA from 200 °C produces CO_x_. The generation of gas leads to voids between the CNTs wrapped on the APP surface, which facilitates the next dispersion. APP decomposes from 275° to produce NH_3_, N_2_ and other non-combustible gases. Afterward, the gas collides with the loose CNTs layer on the surface and incorporates it into NR. Meanwhile, other pyrolysis products of APP, such as polyphosphoric acid, dehydrate the NR into carbon [45].

Secondly, the process of violent combustion of composites is defined as the combustion stage. The internal temperature of the composite material keeps rising. The pyrolysis of TA and APP produces a large amount of refractory gas such as CO_2_, NH_3_, and N_2_, and the high-pressure gas is continuously accumulated inside the composite material. The high-pressure gas extrudes the amorphous carbon produced by the pyrolysis of TA and the pyrolysis of APP so that the amorphous carbon produced by the rubber forms a flame retardant carbonaceous layer. The CNTs are located on the flame-retardant carbonaceous layer and are flushed to the rubber surface by the gas during ignition. CNTs have flame resistance characteristics, a significant aspect ratio, and high strength, which can perfectly support the flame retardant carbonaceous layer. Meanwhile, the O, OH·, R·, and ROO· produced by the pyrolysis of TA and APP trap the free state H· and prevent further expansion of the fire.

Finally, the process of slowly extinguishing the composites is the self-extinguishing stage. The composites have developed two distinct environments. The exterior of the composites comprises open flame and oxygen conditions. The composites inside are combustible, but surrounded by a high concentration of inflammable gas. The boundary between the inside and outside of the material is the flame-retardant carbonaceous layer, supported by CNTs and extruded by high-pressure flame-retardant gas. NR’s combustion process is the flame migration process from outside to inside. The migration process is bound to be hindered by the flame-retardant carbonaceous layer, thereby realizing the self-extinguishing stage of the composite. Figure 8c is the SEM image of the CNTs-supported flame retardant carbonaceous layer in the residue after combustion. The morphology of the CNTs can be clearly seen from the figure, and it can be seen that the CNTs participate in the three stages from combustion to self-extinguishing; CNTs are not burned, and do not destroy their own structure.

### 3.3. Mechanical Properties of NR/CTAPP Composites

**Figure 9:**
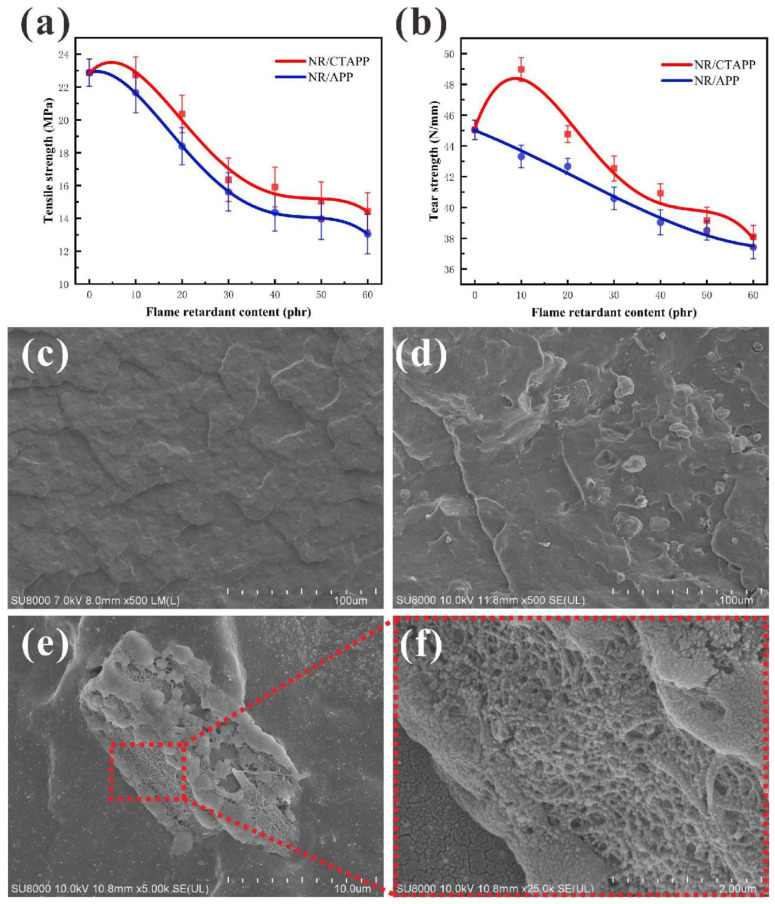
Mechanical properties analysis of composites: (**a**) tensile strength of NR/CTAPP and NR/APP composites; (**b**) tear strength of NR/CTAPP and NR/APP composites; (**c**) low temperature liquid nitrogen brittle fracture section of pure NR; (**d**) low temperature liquid nitrogen brittle fracture section of APP-added composites; (**e**) low temperature liquid nitrogen brittle fracture section of CTAPP-added composites; (**f**) CNTs on the surface of CTAPP flame retardant.

The mechanical properties of rubber directly affect its application environment. The addition of conventional flame retardants leads to a severe decline in the mechanical properties of NR. The tensile strengths shown in Figure 9a,b are those of rubbers with IFR added. It can be seen that the tensile and tear properties of the composites decrease significantly when the amount of flame retardant added increases. This is due to the reduction in the NR content of the cross-section of the composites [46]. As shown in Figure 9c, the section of pure rubber after brittle fracture is smooth, and there are no particle impurities that damage the force surface. As shown in Figure 9d, the cross-section of NR5 after brittle fracture is rough, and there are pits generated by the falling off of the flame retardant and the flame retardant. As a result, the content of rubber with robust mechanical properties on the cross-section of the composites is significantly reduced, and the macroscopic performance is severely decreased in tensile and tear properties. Figure 9e,f is the SEM images of modified CNTs wrapped with ammonium polyphosphate in NR. The NR molding does not destroy the structure of the microencapsulated ammonium polyphosphate. The CNTs are coated on the surface of APP to build a network structure, and then compounded with the NR matrix, which reduces the loss of NR mechanical properties caused by adding APP. The performance gap between NR/CTAPP and NR/APP can also be clearly seen in Figure 9a,b. The tensile and tear properties of the rubber composites both increased slightly at low addition levels. Meanwhile, the performance of NR/CTAPP decreased at high addition levels but is also higher than that of the composites with only APP added. Additionally, Table S1 (Supplementary Materials) shows the variation in volume fractions of NR, CB, and CNTs. The volume fraction of NR decreases from 76.9% for NR0 to 39.4% for NR5 and 39.9% for NR6 as the amount of fillers increases. The combined volume fraction of CB and CNTs, which provide the mechanical properties of NR, decreases from 37.5% for NR0 to 23.7% for NR5 and 32.1% for NR6. In addition to the obvious reason for the decrease in the volume fraction of the NR itself, the decreasing proportion of fillers that contribute to the mechanical properties is also an important factor leading to the decline in the mechanical properties of NR.

## 4. Conclusions

This study uses CNTs to organically combine the carbon source, gas source, and acid source in the intumescent flame retardant system, constructing a special IFR structure of carbon source, gas source, and acid source to enhance the flame retardancy and mechanical properties of NR. CTAPP flame retardant is very sensitive to a combustion reaction and reacts rapidly in the early stage of combustion. The CNTs reinforce the flame-retardant carbonaceous layer, which effectively promotes the internal flame-retardant gas to protect the combustible material from the external flame and the oxygen of the combustion-supporting agent. The modified special intumescent flame retardant effectively replaced the single-component flame retardant, and its performance in LOI, UL-94, and CCT tests all exceeded the latter. When adding 50 phr-modified flame retardant, compared with pure NR, the LOI of the composites increased to 28.6%; PHRR, THR, and TSP decreased by 68.4%, 64.3%, and 49.3%, respectively. The mechanical properties of the composites are slightly improved by low addition amounts. Under high loading levels, it is possible to effectively mitigate the decrease in mechanical properties caused by the addition of flame retardants. Thus, the structure of the intumescent flame retardant in which the carbon source and the carbon forming agent are in close contact is successfully constructed, and the flame retardant mechanism and advantages of the structure are revealed.

## Figures and Tables

**Table 1:** Batch compositions.

Sample	NR Latex	AO	ZnO	SA	Si69	APP	CTAPP	CB	S	CBS
Amounts (phr)	166.7	1.0	5.0	2.0	3.5	Variable	Variable	30.0	1.4	1.2

**Table 2:** Composite formulation and LOI, UL-94 test data.

Sample	Composition			LOI (1%)	UL-94 Rating	Dripping
	NR	APP	CTAPP			
NR0	100	0	0	19.6	NOR	Y
NR1	100	10	-	19.8	NOR	Y
NR2	100	-	10	20.4	NOR	Y
NR3	100	30	-	22.9	V-2	N
NR4	100	-	30	23.7	V-2	N
NR5	100	50	-	27.3	V-1	N
NR6	100	-	50	28.6	V-0	N

NOR: No rating; Y: Yes; N: No.

**Table 3:** Cone calorimetry test data for various samples.

Sample	TTI (s)	PHRR (kM/m^2^)	T_PHRR_ (s)	THR (MJ/m^2^)	TSP (m^2^)	MASS (g)	FPI ^a^ (10^−2^ m^2^s/kW)	FGI ^b^ (kw/m^2^s)	FRI ^c^
NR0	34	1667.26	104	142.06	23.37	4.42	2.04	16.03	1.00
NR1	37	1282.81	116	117.12	23.36	5.94	2.88	11.06	1.72
NR2	39	1119.24	122	134.42	21.97	7.35	3.48	9.17	1.81
NR3	44	1178.39	102	126.15	15.17	8.95	3.73	11.55	2.06
NR4	49	1031.77	119	119.28	13.61	9.96	4.75	8.67	2.77
NR5	52	729.96	83	79.96	12.81	10.75	7.12	8.79	6.21
NR6	58	527.20	90	50.85	11.79	13.74	11.00	5.86	15.07

^a^: FPI = TTI/PHRR. ^b^: FGI = PHRR/T_PHRR_. ^c^: FRI = [THR × PHRR/TTI]_p_/[THR × PHRR/TTI]_c_.

## Data Availability

The data supporting the findings described in this manuscript are available from the corresponding authors upon request.

## Supplementary Materials

The following supporting information can be downloaded at: https://www.mdpi.com/article/10.3390/polym15061406/s1, Table S1: Volume of main fillers of composites.
