# Supplementary material for: Carbon Nanotube-Based Intumescent Flame Retardants Achieve High-Efficiency Flame Retardancy and Simultaneously Avoid Mechanical Property Loss
Source: Polymers (Basel). 2023 Mar 11;15(6):1406. doi: 10.3390/polym15061406 (PMC10056237; doi:10.3390/polym15061406)
Supplement: Supplementary file 1 [file polymers-15-01406-s001.zip › polymers-2238999-supplementary.pdf]

Supplementary Matterials

Carbon Nanotube-Based Intumescent Flame Retardants Achieve High-Efficiency Flame Retardancy and Simultaneously Avoids the Mechanical Property Loss

Qi Qu †, Jin Xu †, Huanhuan Wang, Yinrui Yu, Qianpeng Dong, Xianhua Zhang and Yan He *

Shandong Engineering Laboratory for Preparation and Application of High-performance Carbon Materials, College of Electromechanical Engineering, Qingdao University of Science and Technology, Qingdao, 266061, Shandong, China

* Correspondence: heyan@qust.edu.cn

† These authors contributed equally to this work.

Table S1. Volume of main fillers of composites.

Sample	NR (cm^3)	APP (cm^3)	TA (cm^3)	CB (cm^3)	CNTs (cm^3)	Total filler (cm^3)	NR/Total	(CB+CNT)/Total
NR0	93.0	-	-	55.8	-	148.8	62.5%	37.5%
NR1	93.0	17.4	-	55.8	-	166.2	56.0%	33.6%
NR2	93.0	11.7	1.4	55.8	3.7	165.6	56.1%	35.9%
NR3	93.0	52.2	-	55.8	-	201.0	46.3%	27.8%
NR4	93.0	34.8	4.3	55.8	11.4	199.3	46.7%	33.7%
NR5	93.0	87.0	-	55.8	-	235.8	39.4%	23.7%
NR6	93.0	57.9	7.3	55.8	18.9	232.9	39.9%	32.1%
